# Clinical Case—Rhus Tox

**Published:** 1889-08

**Authors:** H. E. Potter


### CLINICAL CASE.

### RHUS-TOX.

Mrs.——, æt. twenty-two years, had an attack of rheumatism of right shoulder and arm six years ago, and was cured (?) according to orthodox heroic methods. Has suffered more or less ever since till about three months ago, when it became so annoying as to keep her awake three or four nights in succession. She is a homœopath, so I gave her one dose of Rhus-tox$^{200}$ dry, on the tongue, without sac. lac., etc. No symptoms of rheumatism have since shown up; the shoulder is as limber and useful as any other of her joints. The symptoms which led to the selection were a fidgety restlessness, better after having commenced to move and aggravated from cold and at night.

H. E. POTTER, M. D.

